# Meeting the Milestones through Simulation: Disclosing a Medical Error

**DOI:** 10.5070/M5.53010

**Published:** 2026-10-31

**Authors:** Cari Halacoglu, Jessica Andrusaitis

**Affiliations:** * Sutter Roseville Medical Center, Department of Emergency Medicine, Roseville, CA

## Abstract

**Audience:**

This simulation is intended for medical students, interns, junior residents, and senior residents.

**Introduction:**

Patient safety event reporting and disclosure of medical errors to patients and families are critical components of high-quality, transparent care and a necessary skill for emergency medicine (EM) physicians. Accurate reporting of errors, near misses, and unsafe conditions provides institutions with the data needed to investigate vulnerabilities, implement system-based solutions, and prevent recurrence. Disclosure of medical errors fosters trust, supports just culture principles, and reinforces professional accountability.^1^ Physicians have historically reported few adverse events, accounting for only 1–2% of all reports.^2^ To address this gap, the Accreditation Council for Graduate Medical Education (ACGME) now emphasizes patient safety and quality improvement as a part of resident education.

The ACGME Emergency Medicine Milestones have established clear expectations for resident education in patient safety.^3^ Residents, fellows, and faculty must know their responsibilities for event reporting. The milestones include ability to identify patient safety events, understand the system factors that lead to events, and how to complete institutional safety event reports.^3^ ACGME requires residents to participate in real or simulated interprofessional patient safety events and quality improvement activities as part of systems-based practice milestones.^3^ These requirements underscore the importance of experiential learning, feedback, and practice in order to achieve true competence in identifying causes and enacting sustainable safety interventions.

Medication errors, particularly dosing errors, are among the most common patient safety threats in the emergency department (ED).^4^ The ED environment—with high patient volumes, time-sensitive decision-making, and frequent weight-based dosing—places EM physicians at especially high risk for ordering or administering incorrect doses. In this simulation, the wrong concentration of a sedation medication is administered and creates a scenario in which the learner must recognize the error, disclose it to the patient/family, and complete a patient safety report (PSR). This approach integrates clinical management with communication and systems-based practice and provides an avenue for residency programs to facilitate ACGME requirements while preparing residents for real-world patient safety responsibilities in scenarios that otherwise may not yet be encountered during the period of residency.

**Educational Objectives:**

By the end of this simulation activity, learners will be able to: 1) disclose a medical error to a patient and/or their family, 2) understand the different types of patient safety events that should be reported, 3) locate the form for disclosing a patient safety event at their institution, and 4) appropriately complete a patient safety report at their institution.

**Educational Methods:**

The simulation was conducted with a high-fidelity pediatric mannikin. A total of 19 residents and three medical students participated in the simulation during their monthly didactic session. Afterwards, they were guided through completing a patient safety report for their institution.

**Research Methods:**

Learners completed a survey before and after the simulation case assessing their comfort with disclosing medical errors and completing an institutional patient safety report form. The survey questions were formatted in a Likert scale where 1 = strongly disagree, 2 = disagree, 3 = neither agree nor disagree, 4 = agree, 5 = strongly agree. Results collected were analyzed using Wilcox rank-sum test.

**Results:**

The participants completed a survey before and after the experience, and there was a statistically significant improvement in the learners’ comfort level after completing the simulation. The free text responses were also universally positive.

**Discussion:**

Overall, the learners received the simulation well. General feedback was positive; the consensus was that there was increased confidence in patient safety reporting.

**Topics:**

Patient safety, medical error, disclosing error.

## USER GUIDE

**Table:** 

List of Resources:	
Abstract	24
User Guide	26
Instructor Materials	28
Operator Materials	35
Debriefing and Evaluation Pearls	38
Simulation Assessment	41

**Learner Audience:**

Medical students, interns, junior residents, senior residents

**Time Required for Implementation:**

**Preparation:** 20 minutes

**Time for case:** 15 minutes

**Time for debriefing:** 15–20 minutes

**Recommended Number of Learners per Instructor:**

2–4 learners/instructor

**Topics:**

Patient safety, medical error, disclosing error.

**Objectives:**

By the end of this simulation activity, learners will be able to:

1. Disclose a medical error to a patient and/or their family
2. Understand the different types of patient safety events that should be reported
3. Locate the form for disclosing a patient safety event at their institution
4. Appropriately complete a patient safety report at their institution

### Linked objectives and methods

During this simulation, the participants will need to obtain consent from a parent to perform procedural sedation and a fracture reduction in a child. An error will be made in dosing the sedation medication for the child, and the participants will need to disclose this to the family (objective 1). In the debrief, they will receive education on which types of things should be reported as patient safety events (objective 2). Lastly, they will be guided through filling out a patient safety report (objectives 3 and 4).

### Recommended pre-reading for instructor

Review your institution’s mechanism to report a safety event or medical error.

### Results and tips for successful implementation

This simulation is best implemented in a realistic environment with a high-fidelity mannikin that gives real time information like changing vital signs. Best group sizes are four to six. An embedded participant that can act as the parent is necessary.

A total of 21 participants completed the simulation: 19 emergency medicine residents and three medical students. All participants completed a pre- and post-simulation survey using a 5-point Likert scale and free response. The questions were aimed at assessing the learners’ comfort level with our objectives. Survey questions assessed their comfort level with identifying different types of medical errors that should be reported, communicating these errors with patients and their families, and their confidence in how to locate and complete a patient safety error report at their institution. Learners scored statistically significantly higher (*P*<.05) on all questions after the simulation when compared with before the simulation (Figure 1). Qualitative feedback was positive overall. Multiple learners specifically commented that they previously had had no exposure to patient safety reporting and thus found this experience very helpful: “especially helpful with pediatric patient scenario” and “I’m glad we completed a PSR in association with this sim case; it helped solidify the importance of error reporting and conversations with parents, especially with a pediatric patient.” Comments also included feedback that this case emphasized the importance of drawing up your own sedation medications and confirming dosages prior to administering them.

The critical action to obtain informed consent was included in this simulation because it flows naturally with the clinical scenario presented. However, for the sake of time, we chose not to emphasize this exercise in the simulation objectives because obtaining informed consent is a scenario that presents more frequently in real clinical practice, and we anticipate there are other avenues for evaluating and providing feedback for residents on this skill. Allow for more time in debriefing if choosing to include informed consent as an objective in this activity when executed.

## Figures and Tables

**Table:** 

**#1**	**X-ray of left forearm**
**#2**	**EKG**

**Table:** 

**Vitals:**	Heart Rate (HR) 101	Blood Pressure (BP) 115/72
	Respiratory Rate (RR) 21	Temperature (T) 37.5°C
	Oxygen Saturation (O_2_Sat) 99% on room air (RA)	

**Figure:**
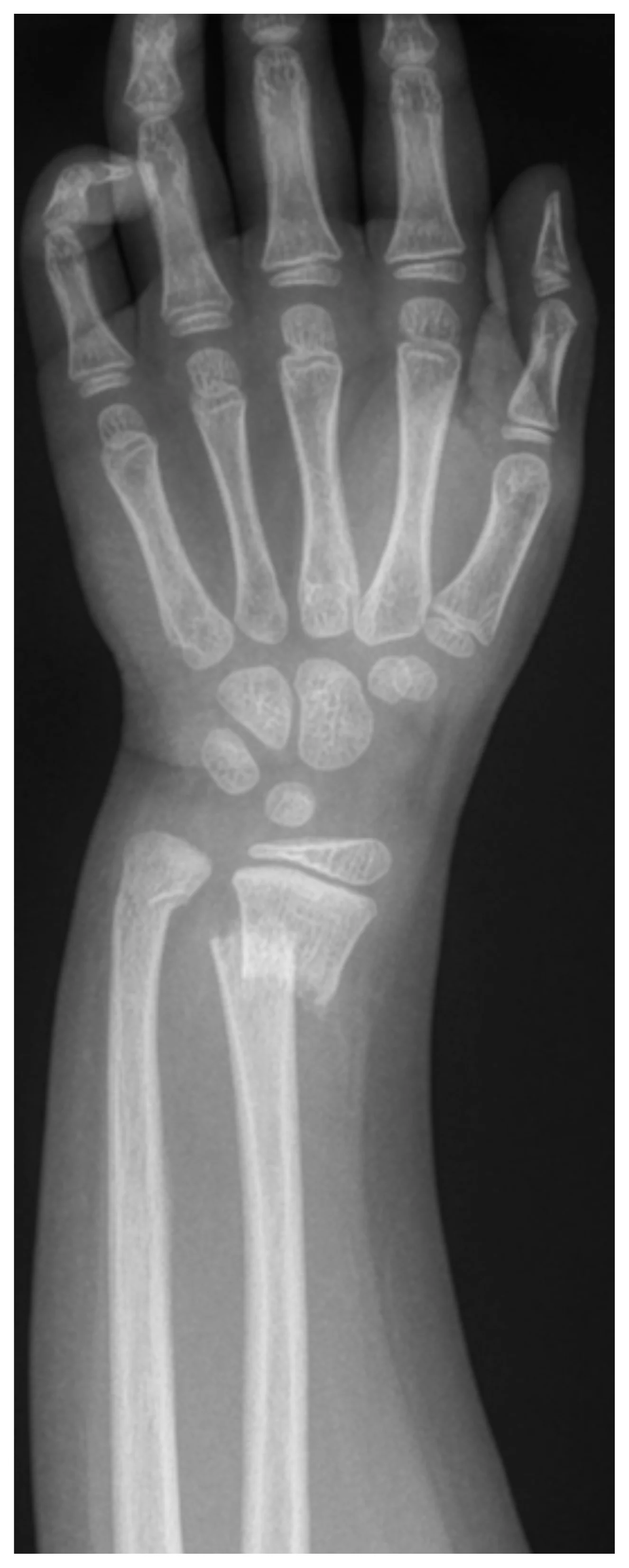


**Figure:**
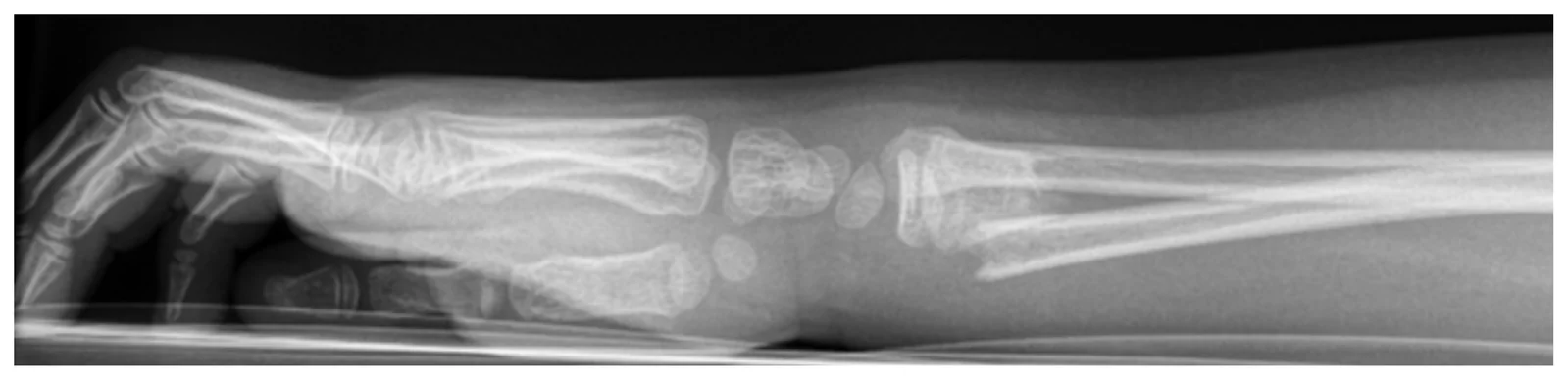


**Figure:**
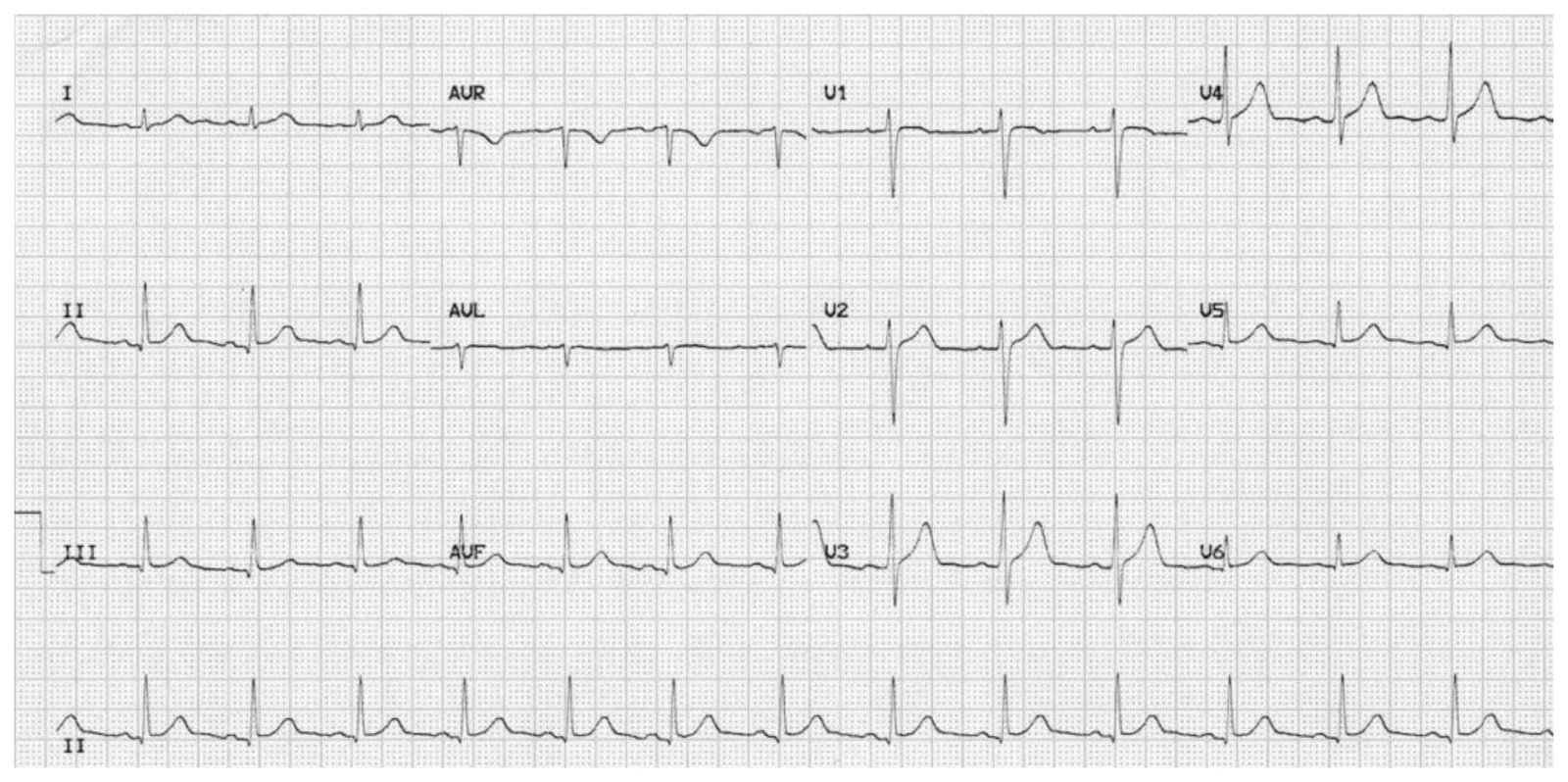


**Table:** SIMULATION EVENTS TABLE:

Minute (state)	Participant action/ trigger	Patient status (simulator response) & operator prompts	Monitor display (vital signs)
0:00 (Baseline)	Learner should obtain vital signs and place IV. Perform trauma survey and the history and physical. Order workup (X-ray).	Patient is awake and alert but in pain; intermittent crying especially if left arm is touched.	Rhythm: normal sinus (NS) HR: 108 BP: 115/72 RR: 21 SPO2: 99% T:37.5 °C
2:00	Learner recognizes the need for procedural sedation for displaced fracture reduction and discusses procedure plan. Optional objective: obtain brief informed consent from parent. .	If any other medication than ketamine is chosen, redirect the learner to ketamine. (eg, “there is a national shortage of that medication,” or parent can say, “I don’t want him to have that one. He had a reaction last time he broke his arm.”)	HR: 120 BP: 115/72 RR: 21 SPO2: 99%
4:00 Sedation prep	Set up all sedation equipment. Order appropriate ketamine dose, which the nurse will incorrectly prepare.	Nurse should obscure the medication error by stepping out to “get the ketamine from the pyxis.” They will mistakenly draw up 3.5–5 ml of the intramuscular (IM) concentration 500 mg/10 ml (50 mg/ml) and not the IV concentration 200 mg/20 ml (10 mg/ml).	HR: 120 BP: 115/72 RR: 21 SPO2: 99%
5:00 Sedation starts	Time out called. Incorrect dose of ketamine given.	Patient’s oxygen desaturates after the large dose of ketamine is administered rapidly.	HR: 108 BP: 127/80 RR: 14 SPO2: 86%
6:00 Decompensation	Learner should address the desaturation (bag valve mask, intubate, etc.).	If nothing is done for hypoxia, it will worsen, and patient will ultimately continue to decompensate, requiring intubation for airway protection. If ventilating with BVM, the patient slowly improves.	No action: HR: 94 BP: 90/56 RR: 8 SPO2: 72% BVM: HR: 100 BP: 100/60 RR: 16 SPO2: 91%
7:00 Sedation ends Error aftermath	Nurse informs the team of the error.	Patient starts to moan and wake up or is intubated. Parent re-enters the room, is visibly and verbally upset when they see the child requiring airway interventions. (eg, “What happened?! What did you do to my child? You said this was a safe and routine procedure and that he would still be breathing on his own!”)	Not intubated: HR: 110 BP: 122/80 RR: 20 SPO2: 99% Intubated: HR: 120 BP 115/72 RR: 16 SPO2: 99% on vent
Case Completion	Learner discloses error to parent. Learner completes a PSR.	Nurse may need to prompt learner to disclose and report this. (eg, “Shouldn’t we tell the parent what happened?” “Isn’t there some way this mistake can be reported so it doesn’t happen again?”)	HR: 120 BP: 115/72 RR: 20 SPO2: 99%

**Table:** 

**Critical Actions:** Devise sedation plan to reduce distal radius fracture Obtain informed consent from parent for the procedural sedation Appropriately address the hypoxia during the sedation (i.e., bag valve mask, intubation, etc.) Disclose the error to the patient’s family member Complete a PSR through the learner’s institution	0:00

**Table:** 

	Milestone	Did not achieve level 1	Level 1	Level 2	Level 3
1	**Emergency Stabilization (PC1)**	□ Did not achieve Level 1	□ Recognizes abnormal vital signs	□ Recognizes an unstable patient, requiring intervention Performs primary assessment Discerns data to formulate a diagnostic impression/plan	□ Manages and prioritizes critical actions in a critically ill patient Reassesses after implementing a stabilizing intervention
2	**Performance of focused history and physical (PC2)**	□ Did not achieve Level 1	□ Performs a reliable, comprehensive history and physical exam	□ Performs and communicates a focused history and physical exam based on chief complaint and urgent issues	□ Prioritizes essential components of history and physical exam given dynamic circumstances
3	**Diagnostic studies (PC3)**	□ Did not achieve Level 1	□ Determines the necessity of diagnostic studies	□ Orders appropriate diagnostic studies. Performs appropriate bedside diagnostic studies/procedures	□ Prioritizes essential testing Interprets results of diagnostic studies Reviews risks, benefits, contraindications, and alternatives to a diagnostic study or procedure
4	**Diagnosis (PC4)**	□ Did not achieve Level 1	□ Considers a list of potential diagnoses	□ Considers an appropriate list of potential diagnosis May or may not make correct diagnosis	□ Makes the appropriate diagnosis Considers other potential diagnoses, avoiding premature closure
5	**Pharmacotherapy (PC5)**	□ Did not achieve Level 1	□ Asks patient for drug allergies	□ Selects an medication for therapeutic intervention, consider potential adverse effects	□ Selects the most appropriate medication and understands mechanism of action, effect, and potential side effects Considers and recognizes drug-drug interactions
6	**Observation and reassessment (PC6)**	□ Did not achieve Level 1	□ Reevaluates patient at least one time during case	□ Reevaluates patient after most therapeutic interventions	□ Consistently evaluates the effectiveness of therapies at appropriate intervals
7	**Disposition (PC7)**	□ Did not achieve Level 1	□ Appropriately selects whether to admit or discharge the patient	□ Appropriately selects whether to admit or discharge Involves the expertise of some of the appropriate specialists	□ Educates the patient appropriately about their disposition Assigns patient to an appropriate level of care (ICU/Tele/Floor) Involves expertise of all appropriate specialists
9	**General Approach to Procedures (PC9)**	□ Did not achieve Level 1	□ Identifies pertinent anatomy and physiology for a procedure Uses appropriate Universal Precautions	□ Obtains informed consent Knows indications, contraindications, anatomic landmarks, equipment, anesthetic and procedural technique, and potential complications for common ED procedures	□ Determines a back-up strategy if initial attempts are unsuccessful Correctly interprets results of diagnostic procedure
20	**Professional Values (PROF1)**	□ Did not achieve Level 1	□ Demonstrates caring, honest behavior	□ Exhibits compassion, respect, sensitivity and responsiveness	□ Develops alternative care plans when patients’ personal beliefs and decisions preclude standard care
22	**Patient centered communication (ICS1)**	□ Did not achieve level 1	□ Establishes rapport and demonstrates empathy to patient (and family) Listens effectively	□ Elicits patient’s reason for seeking health care	□ Manages patient expectations in a manner that minimizes potential for stress, conflict, and misunderstanding. Effectively communicates with vulnerable populations, (at risk patients and families)
23	**Team management (ICS2)**	□ Did not achieve level 1	□ Recognizes other members of the patient care team during case (nurse, techs)	□ Communicates pertinent information to other healthcare colleagues	□ Communicates a clear, succinct, and appropriate handoff with specialists and other colleagues Communicates effectively with ancillary staff

**Figure 1:**
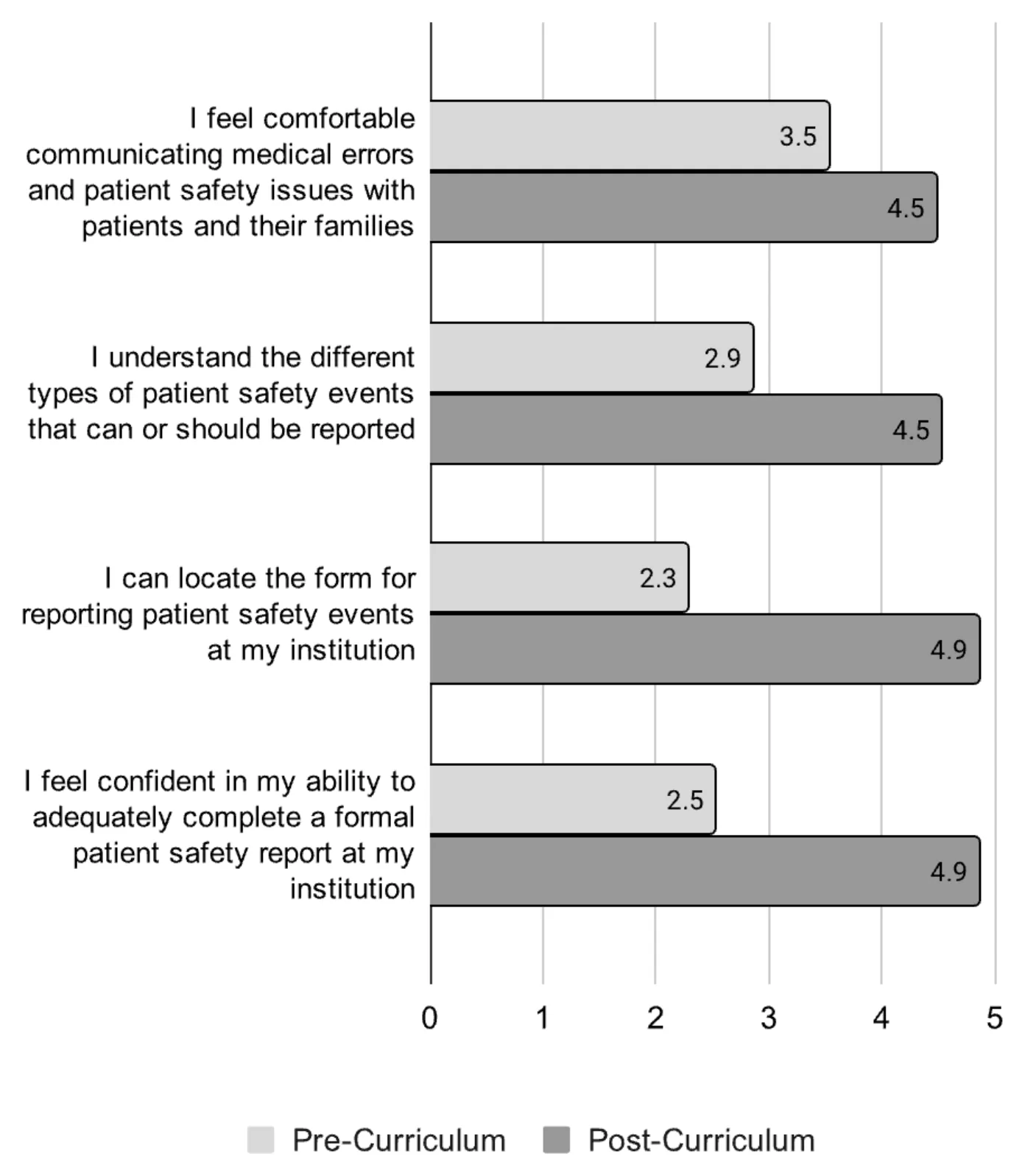
Pre- and Post-Curriculum Survey Responses

## INSTRUCTOR MATERIALS

**Case Title:** Meeting the Milestones through Simulation: Disclosing a Medical Error

**Case Description & Diagnosis (short synopsis):** This scenario presents a 6-year-old male who fell while skateboarding and injured his left arm. A parent brings him to the ED for evaluation. Participants perform a trauma evaluation and determine the child has sustained a displaced left distal radius fracture that will require sedation for reduction. After consent is obtained from the parent, the sedation procedure takes place, but an error is made in the dosing of the medication. The participants must disclose to the parent that an error was made and then appropriately report the safety event.

**Equipment or Props Needed:**

- High fidelity mannikin (we used a pediatric one)
- Vital signs monitoring
- Oxygen delivery supplies
- Suction
- Pediatric resuscitation cart with airway supplies (aka Broselow cart)
- Simulated medications: ketamine vials in two different concentrations (200 mg/20 ml [10 mg/ml] and 500 mg/10 ml [50 mg/ml])
- Splinting material
- Computer workstation for learners to complete PSR

**Embedded participants needed:**

Assistants to play patient’s parent/family member and procedural sedation support staff such as bedside nurse and/or ED pharmacist.

**Stimulus Inventory:**

**Background and brief information:** This scenario takes place at an urban emergency department. The patient is a 6-year-old male brought in by private vehicle driven by parent.

**Initial presentation:** On initial presentation, the child is lying in bed intermittently whimpering in pain and holding his left arm.

**How the scenario unfolds:** The scenario presents a 6-year-old male who fell while skateboarding and is brought to the emergency department for left arm pain by his parent. The initial evaluation should include obtaining history, performing trauma evaluation, putting the child on the monitor, and ordering a workup (i.e., X-ray of the left arm).

The X-ray will show a displaced left distal radius fracture which should prompt the team to perform procedural sedation for fracture reduction and splinting. There are no other injuries and the patient remains stable. The participants will need to devise a sedation plan and obtain consent from the parent. The instructor needs to guide the participants towards using ketamine. If a medication other than ketamine is selected, the parent can refuse based on a bad prior reaction, parental concern, or the pharmacy or nurse can say there is a national shortage of the alternative selected medication.

During the sedation, the nurse will give the incorrect dose of ketamine. The participants should correctly ask for 1–1.5 mg/kg of intravenous (IV) ketamine, but the nurse will mistakenly draw up 3.5–5 ml of the intramuscular (IM) concentration 500mg/10ml (50mg/ml) and not the IV concentration 200mg/20ml (10mg/ml). The nurse should have this already drawn up in a pocket or step outside to get it, instead of drawing it up inside the room just in case the learners ask to watch or do it themselves. Instead of receiving 35–50 mg of IV ketamine, the patient receives 175–250 mg of IV ketamine. The patient’s oxygen saturation level will decrease, and the participants should react to this decompensation by providing bag valve mask ventilation to the patient and perform a jaw thrust at minimum.

Afterwards, the nurse should “discover” the medication error that was made and notify the learner. The team should disclose the error to the parent and report the safety event. This may require prompting from the nurse. Filling out the Patient Safety Report document should be done either during the case or immediately after during debrief.

**Critical actions:**

1. Devise sedation plan to reduce distal radius fracture
2. Obtain informed consent from parent for the procedural sedation
3. Appropriately address the hypoxia during the sedation (i.e., bag valve mask, intubation, etc.)
4. Disclose the error to the patient’s family member
5. Complete a PSR through the learner’s institution

**Case Title:** Meeting the Milestones through Simulation: Disclosing a Medical Error

**Chief Complaint:** Left arm pain and deformity after fall

**General Appearance:** Non-toxic, well-appearing male child, holding onto left arm.

**Primary Survey:**

- **Airway:** Patent. Speaking in full sentences.
- **Breathing:** Breath sounds bilaterally, normal respiratory rate.
- **Circulation:** Warm, well perfused, pulses intact. No bleeding.

**History:**

- **History of present illness:** Left arm pain after falling on a skateboard 1 hour prior to arrival. Tried to brace fall with an outstretched arm, immediately felt pop and pain. Came into the house crying and bracing his arm; the event was not witnessed by parents. He was wearing a helmet. No loss of consciousness or evidence of head injury. Denies headache, neck pain, nausea, or vomiting.
- **Past medical history:** Right arm fracture 3 years ago.
- **Past surgical history:** No surgical history.
- **Patient’s medications:** No current medications.
- **Allergies:** No known allergies.

**Secondary Survey/Physical Examination:**

- **General appearance:** Non-toxic, well-appearing male child, holding onto left arm.
- **HEENT:** Clear oropharynx. Within normal limits.
- **Neck:** Within normal limits.
- **Heart:** Within normal limits.
- **Lungs:** Within normal limits.
- **Abdominal/GI:** Within normal limits.
- **Genitourinary:** Within normal limits.
- **Extremities:** Gross deformity left distal forearm. No wounds. Motor function and sensation intact in distal extremity. Radial pulses intact bilaterally. Soft, compressible compartments in extremity. Brisk capillary refill.
- **Back:** Within normal limits.
- **Neuro:** Within normal limits.
- **Skin:** Within normal limits.
- **Lymph:** Within normal limits.
- **Psych:** Within normal limits.

*X-ray of Left Forearm*

Chan A. Distal radius and ulna fractures. In: Radiopaedia.org. https://doi.org/10.53347/rID-178324. Accessed May 22, 2026. CC-NC-BY-SA 3.0.

*EKG*

Normal sinus rhythm EKG. Life In The Fast Lane. https://litfl.com/wpcontent/uploads/2018/08/normal-sinus-rhythm-2.jpg. Accessed October 1, 2025. CCBY-SA 4.0.

Labs are not necessary for this case. Should the learner order labs, report that they are all within normal limits.

## OPERATOR MATERIALS

**Diagnosis:** Closed fracture of left distal radius with sedation that results in a medical error

**Disposition:** Discharge if near miss event; admitted if escalated to intubation.

## DEBRIEFING AND EVALUATION PEARLS

### Disclosing a Medical Error

**Pearls:**

Medical errors are preventable events that occur during the delivery of healthcare and can involve incorrect diagnoses, treatment delays, communication failures, or medication mistakes. Medical errors contribute to tens of thousands of preventable deaths each year in the U.S.^1^ Emergency departments are particularly high-risk, with medication errors affecting nearly 20% of patients.^4^ Reporting patient safety events allows healthcare systems to identify risks, learn from mistakes, and implement changes that improve care. Electronic reporting systems make it easier to collect and analyze this data, helping institutions address systemic issues rather than isolated incidents.^2^

**Types of safety events to report:**

- **Near Miss:** An error that is caught before reaching the patient (eg, a wrong medication dose intercepted).
- **Adverse Event:** An injury caused by medical care, regardless of error (eg, an allergic reaction).
- **Medical Error:** A failure in planning or execution of care (eg, administering the wrong medication).
- **Sentinel Event:** A serious, unexpected event involving death or major harm; requires immediate investigation (ex. wrong side or site during surgery).

**Just Culture:**

A “just culture” recognizes that most errors are not caused by reckless behavior, but by system flaws or human fallibility. It encourages reporting by distinguishing between human error, at-risk behavior, and intentional misconduct. This approach promotes learning over punishment and supports continuous improvement.^5^

**Creating a Culture of Safety:**

A strong safety culture is one where all healthcare team members feel safe to speak up about concerns. Organizations that promote transparency, non-punitive feedback, and leadership accountability tend to have better reporting rates and patient outcomes.^5^

**Disclosing Errors to Patients and Families:**

When an error occurs, honest and empathetic communication is essential. Patients want to know what happened, how it will affect their care, and what will be done to prevent it from happening again. Clear disclosure builds trust and reinforces ethical, patient-centered care. 6

Before the Conversation: Prepare

- Review the event with team members — ensure clarity on what happened if possible
- Decide which team members should be present, who will speak, and what will be said
- Anticipate likely questions and patient/family emotional responses
- Prepare to listen actively and validate their feelings
- Plan for follow-up conversations and share contact information

During the Conversation: What to Say

- Use simple language — no medical jargon
- Share the known facts clearly and calmly
- Explain:
  ○ What is being done right now for the patient
  ○ How the event may impact their overall care
  ○ What is being done to prevent future harm
  ○ Offer a genuine apology — no excuses, no “but”
  ○ Example: “I am truly sorry this happened. We are taking this very seriously.”
- Answer all questions honestly and respectfully
- Emphasize that it is not the last conversation regarding the incident

After the Conversation:

- Debrief with your team
- Document the disclosure in the medical record:
  ○ Keep it brief and objective (eg, “Spoke with patient/family regarding adverse event. Explained facts and current plan. Expressed regret. All questions answered.”)
- Submit a Patient Safety Report (PSR)

**Other debriefing points:**

Debriefing can include a review of the components of informed consent and be an opportunity for providing resident feedback to solidify this skill, which can optionally be added as an objective to this simulation activity if time permits. Debriefing should provide feedback on the effectiveness of the learner’s overall communications skills during challenging conversations, particularly with disclosing medical errors that could cause harm.

Prior to presenting this simulation activity, we met with our institution's safety and risk management department to obtain their recommendations on how to approach disclosing a medical error and the internal processes that occur after a report is made. Sharing this information with residents can help convey the culture of safety at your institution and instill trust in the processes, which may be a barrier to reporting.

**Wrap Up:**

We suggest providing slides and a printed handout with instructions to complete a patient safety event report. The instructions include multiple computer screenshots of where to locate the electronic form and further explains in a step-by-step manner how best to ensure the form is submitted properly. This resource was omitted from our submission for institutional security reasons but resources can be created for your specific institution.

## SIMULATION ASSESSMENT

*Meeting the Milestones through Simulation: Disclosing a Medical Error*

Learner: _________________________________________

***Assessment Timeline***

This timeline is to help observers assess their learners. It allows observer to make notes on when learners performed various tasks, which can help guide debriefing discussion.

**Critical Actions:**

□ Devise sedation plan to reduce distal radius fracture
□ Obtain informed consent from parent for the procedural sedation
□ Appropriately address the hypoxia during the sedation (i.e., bag valve mask, intubation, etc.)
□ Disclose the error to the patient’s family member
□ Complete a PSR through the learner’s institution

**Summative and formative comments:**

**Milestones assessment:**
